# The association of management and leadership competencies with work satisfaction among pharmacists in Lebanon

**DOI:** 10.1186/s40545-023-00554-z

**Published:** 2023-03-21

**Authors:** Lama Zeineddine, Hala Sacre, Chadia Haddad, M. Rony Zeenny, Marwan Akel, Aline Hajj, Pascale Salameh

**Affiliations:** 1 Faculty of Pharmacy, Lebanese University, Hadat, Lebanon; 2 INSPECT-LB (Institut National de Santé Publique, d’Épidémiologie Clinique et de Toxicologie-Liban), Beirut, Lebanon; 3 School of Medicine, Lebanese American University, Beirut, Lebanon; 4 Research Department, Psychiatric Hospital of the Cross, P.O. Box 60096, Jal Eddib, Lebanon; 5 School of Health Sciences, Modern University for Business and Science, Beirut, Lebanon; 6 Department of Pharmacy, American University of Beirut Medical Center, Beirut, Lebanon; 7 School of Pharmacy, Lebanese International University, Beirut, Lebanon; 8 Laboratoire de Pharmacologie, Pharmacie Clinique et Contrôle de Qualité des Médicament (LPCQM), Faculty of Pharmacy, Saint-Joseph University of Beirut, Beirut, Lebanon; 9 Faculté de Pharmacie, Université Laval, Québec, Canada; 10 Department of Primary Care and Population Health, University of Nicosia Medical School, 2417 Nicosia, Cyprus; 11 Oncology Division, CHU de Québec Université Laval Research Center, Québec, Canada

**Keywords:** Competency, Leadership, Management, Pharmacist, Work satisfaction

## Abstract

**Background:**

Pharmacists are at the core of the healthcare system and are the most accessible healthcare professionals. Their new roles involve leadership skills, among others. Work satisfaction of pharmacists might affect the quality of the services they provide. Hence, the primary objective of this study was to evaluate the management/leadership skills and work satisfaction of pharmacists and working pharmacy students. The secondary objective was to establish the relationship between management/leadership competencies and work satisfaction.

**Methods:**

This cross-sectional study enrolled 415 Lebanese pharmacists and fifth-year pharmacy students (undergraduates) working in different pharmacy sectors across Lebanon from August 2021 through October 2021 using the snowball sampling technique and validated tools to assess management/leadership competencies and work satisfaction.

**Results:**

Management/leadership competencies were significantly correlated with work satisfaction (*B* = 0.288) and inversely associated with being engaged/married (*B* = − 2.825) and living outside Beirut or Mount Lebanon (*B* = − 1.873). Pharmacy students did not significantly differ in their leadership/management level from graduate pharmacists. Work satisfaction was significantly associated with management/leadership competencies (*B* = 0.062) and inversely related to education level (*B* = − 0.644).

**Conclusions:**

Pharmacists’ work satisfaction and management/leadership competencies are interrelated, although the level of satisfaction seemed lower than the declared level of competencies. These concepts are differentially affected by personal and work-related characteristics. More efforts should be exerted to improve both the satisfaction and management/leadership competencies of pharmacists in Lebanon.

## Introduction

Leadership is about doing the right things, whereas management is about doing things right [1]. Even if there are some differences between leadership and management, usually, these two skills overlap [1]. Successful leaders can also be skilled managers, and many managers have leadership qualities. However, while leaders are concerned with the organization’s mission or vision, managers are concerned with accomplishing that mission or vision [1]. Thus, leaders get people excited about doing the work, and managers ensure achieving more operational details to get it done.

Pharmacists are at the core of the healthcare system and are the most accessible healthcare professionals [2]. Their essential role is compounding and dispensing safe and effective medications. However, over the years, the pharmacy practice has evolved and become more patient-centered and outcome-oriented, seeking to optimize patient and medication therapy outcomes. It now includes patient education, patient counseling, providing drug information, and monitoring drug therapy, in addition to the technical aspects of pharmaceutical services [3].

Pharmacy leaders should maintain their practice skills, keep up with new technology, think strategically, understand the organization’s finances, and manage supplies well. They are responsible for promoting the enthusiasm that supports and enhances productivity through mobilizing resources, taking opportunities, inspiring creativity, and empowering others. Leaders are acknowledged for their self-awareness, determination, courage, flexibility, creativity, objectivity, and ethical behavior [4]. They also have curiosity, acceptance, warmth, and respect for differences between themselves and individual employees [5]. Thus, forming successful leaders is imperative to the pharmacy profession [5].

Evolving as an efficient manager is also one of the essential traits needed for pharmacists, who should be able to manage different kinds of resources while managing their staff [6]. They should also ensure the quality of medications and improve their clinical competencies in patient care activities [6]. Pivotal components for the quality of pharmaceuticals and care are developing and maintaining department policies, goals, objectives, quality assurance programs, and safety, environmental, and infection control standards [6].

Furthermore, work satisfaction is a multidimensional, enduring, and much sought-after concept in organizational behavior, as it is closely related to job happiness [1]. It is an outcome affected by intrinsic factors, including achievement, recognition, responsibility, and continuing education, or extrinsic factors, such as quality of the work environment, interpersonal relations, and professional interactions. In contrast, other factors causing stress, such as excessive workload, could be against work satisfaction [7–9]. Work satisfaction is also related to productivity [10]. Consequently, it directly affects labor market behavior and economic efficiency, while its lack can lead to stress, burnout, and a decrease in job performance, engagement, and commitment [11, 12]. Thus, satisfaction is a fundamental requirement for the effective functioning of any professional practice.

Assessing work satisfaction among pharmacists is paramount to give insight into the quality of pharmaceutical services and generate information that could change pharmacists’ approach to patient care and increase customer satisfaction [13]. Therefore, unveiling the area, where pharmacists show a low satisfaction level can help put in place measures to improve it. Lack of leadership causes disengagement, making change even more difficult [4]. Pharmacists with leadership abilities should be identified and placed in positions, where they can succeed. Both effective leadership and the work satisfaction of the employee contribute to the success of the organization.

Many worldwide studies have explored leadership or job satisfaction among pharmacists, but only a few linked personal leadership competencies to work satisfaction: a global study reported an inverse association between job expectation and satisfaction [14]. A counter-example, showed that in Texas, some types of leadership could be associated with job satisfaction among retail pharmacists [15]. Thus, there is still a need to assess this association.

This study is the first in Lebanon to assess the management/leadership competencies and their association with work satisfaction among a sample of working Lebanese pharmacists and fifth-year pharmacy students. It was conducted in a context of sanitary and economic challenges; in October 2019, the country went through multiple crises, starting with political turmoil, the COVID-19 pandemic, and a severe socioeconomic hardship that led to the devaluation of the Lebanese currency by more than 95%. This situation affected the health sector in general and the pharmaceutical sector in particular and shed its burden on healthcare professionals, including pharmacists, in terms of burnout and work dissatisfaction [16].

The primary objective of this study was to evaluate the management/leadership skills and work satisfaction of pharmacists and pharmacy students. The secondary objectives were to establish the relationship between management/leadership competencies and work satisfaction and assess the impact of various variables on management/leadership competencies and work satisfaction.

## Methods

### Study design and target population

An observational cross-sectional study was conducted among 415 Lebanese pharmacists and fifth-year pharmacy students (undergraduates) working in different pharmacy sectors across Lebanon from August 2021 through October 2021. It should be noted that in Lebanon, fifth-year pharmacy students are allowed to work, because, at that time, they would have already completed the 12-month internship required by the Order of Pharmacists of Lebanon, the official pharmacists’ association [17].

The inclusion of participants was based on a snowball technique selection, regardless of their years of experience, gender, age, marital status, education level, and region. It was anticipated that this diversity would improve the representativeness of the sample to the entire population of pharmacists and pharmacy students in Lebanon. Due to practical difficulties in conducting face-to-face interviews, including COVID-19-related movement restrictions and the severe socioeconomic crisis that has increased the cost of transportation, thereby hindering travel between different geographic regions, the questionnaire was available online. It was first shared with a group of pharmacists/pharmacy students, who, in turn, helped enroll future participants. The only inclusion criterion considered was “Lebanese pharmacists/pharmacy students currently working”. Participation in the study was voluntary, and the data were collected anonymously. Participants received no financial rewards in exchange for their participation.

### Sample size

The Epi-Info 7 software version 7.2.4.0 was used to calculate a minimum sample of 384 participants, based on the assumption that the probability of having management/leadership competencies or being satisfied at work in the target population is 0.5 (50%) at a 95% confidence interval (limit of precision 5% with a design effect of 1). The *P* = 50% assumption was decided to ensure a sufficient sample size, because no available estimate for *P* was found in the literature about the prevalence of these competencies among Lebanese pharmacists and pharmacy students. Accordingly, the survey portal was closed, and data collection was stopped when the number exceeded the sample size.

### Data collection tool

The questionnaire included 33 questions divided into three main sections to cover the impact of management/leadership competencies on work satisfaction.

The first section included sociodemographic data, such as age, gender, marital status, education level, school/faculty of pharmacy, region, work sector, years of experience, employment type, and monthly income.

The second section was related to management/leadership competencies. It included the Leadership Skills Questionnaire, a validated scale adapted from “*Leadership, Theory, and Practice by Peter G. Northouse”* [18]. This 18-item tool measures three types of leadership skills of 6 questions each, i.e., administrative (items 1, 4, 7, 10, 13, and 16), interpersonal (items 2, 5, 8, 11, 14, and 17), and conceptual (items 3, 6, 9, 12, 15, and 18). All items were graded on a 5-point Likert scale from 1 (Not true) to 5 (Very true). After summing up, a higher score indicates a higher leadership.

The third section consisted of the Short Index of Job Satisfaction (SIJS) used to assess work satisfaction. It is a 5-item self-report psychometric instrument created by Brayfield and Rothe [19], where participants respond to items rated on a 5-point scale as follows: 1 (Strongly Disagree), 2 (Disagree), 3 (Undecided), 4 (Agree), 5 (Strongly Agree), with items 3 and 5 being reversed. The total score was calculated by summing up all the items. The overall job satisfaction score for each participant falls between 5 and 25, with no cutoff score, where higher scores indicate better job satisfaction. The scales were used after getting permission from the respective authors.

Since the great majority of pharmacists and undergraduates speak English in Lebanon, no translation or cultural adaptation of questionnaires was deemed necessary. A pilot-testing was applied after structuring the questionnaire and the respondents agreed that the questions and layout were clear enough and did not need any improvement. The overall time it took to fill the questionnaire is 3–5 min. Data from the pilot study were not included in the final database.

### Statistical analysis

The data were analyzed using the statistical software SPSS (Statistical Package for Social Sciences) version 25. A descriptive analysis was performed, where categorical variables were expressed as absolute frequencies and percentages and quantitative variables as means and standard deviations. The qualitative variables were gender, marital status, education level, school/faculty of pharmacy, region, work sector, employment type, years of experience, and monthly income. The quantitative variables were job satisfaction, leadership, and age.

The reliability of the scales was performed, considering the internal consistency. The Cronbach’s alpha coefficient for the management/leadership competencies value was 0.982, and for work satisfaction, it was 0.33.

For bivariate analysis, parametric tests were used after checking the normality of the continuous variables using visual inspection, skewness and kurtosis, since the sample size was higher than 300. Consequently, the independent-sample *t* test was used to compare the mean between two groups, whereas the ANOVA test was used to compare three or more means.

A multiple linear regression analysis using the forward method was performed to predict job satisfaction and management/leadership competencies. All the baseline variables that showed significance in the bivariate analysis (*p* value < 0.2) were included in the model; this selection was performed to account for all potential confounders. All the variance inflation factors (VIF) were below 10; thus, there was no collinearity between the variables in the model. Variables retained in the models were those that had a significant association with the dependent variables, while those with no significant association were excluded. In all cases, a *p* value < 0.05 was considered significant.

## Results

### Sociodemographic characteristics

The majority of the participants were females (72.5%; *n* = 301), single (76.4%; *n* = 317), and residing in Mount Lebanon and Beirut (69.6%; *n* = 289). Of the total sample, 22.4% were fifth-year (undergraduate) (*n* = 93), 28% had a bachelor's degree (*n* = 115), 18.1% had a master’s degree (*n* = 124), and 17.6% were Doctor of Pharmacy (PharmD) (*n* = 73). The mean age of participants was 25.97 years, with a minimum of 19 and a maximum of 59 years (Table 1).

**Table 1:** Demographic characteristics of the participants

Characteristic	*n* %
Gender, *n* = 415	
Females	301 (72.5)
Males	114 (27.5%)
Marital status, *n* = 415	
Single	317 (76.4)
Married-Engaged	95 (22.9)
Divorced	3 (0.7)
Highest education level, *n* = 415	
Undergraduate	93 (22.4)
Bachelor’s Degree	115 (27.7)
Master’s Degree	75 (18.1)
PharmD	73 (17.6)
PharmD, Master’s Degree	49 (11.8)
PharmD, PhD	9 (2.2)
PhD	1 (0.2)
School/faculty of pharmacy, *n* = 415	
Beirut Arabic University (BAU)	49 (11.8)
Lebanese American University (LAU)	40 (9.6)
Lebanese International University (LIU)	57 (13.7)
Lebanese University (LU)	216 (52)
Saint-Joseph University of Beirut (USJ)	40 (9.6)
Outside Lebanon	13 (3.1)
Region, *n* = 415	
Beirut	101 (24.3)
Beqaa	28 (6.7)
Mount Lebanon	188 (45.3)
Nabatiyeh	11 (2.7)
North	35 (8.4)
South	52 (12.5)
Beirut	101 (24.3)

### Work characteristics

All respondents were Lebanese pharmacists or pharmacy students who are currently working, based on the inclusion criterion. The highest proportion of participants (65.3%; *n* = 271) worked in a community pharmacy, and the majority of participants (83.9%; *n* = 348) had practiced for less than 5 years. More than half of the participants (54%; *n* = 224) worked full-time, and the remaining 46% (*n* = 191) were part-timers. Regarding income, 178 (43%; *n* = 178) earned less than 1.500.000 L.L. per month (Table 2).

**Table 2:** Work characteristics of the participants

Work characteristics	*n* %
Work sector, *n* = 415	
Community pharmacy	271 (65.3)
Pharmaceutical Company	60 (14.5)
Hospital	27 (6.5)
Industry	9 (2.2)
Academia	17 (4.1)
More than one work sector	31 (7.5)
Years of experience, *n* = 415	
≤ 5	348 (83.9)
[6–10]	35 (8.4)
[11–15]	17 (4.1)
[16–20]	6 (1.4)
> 20	9 (2.2)
Employment type, *n* = 415	
Part-time	191 (46)
Full-time	224 (54)
Monthly income, *n* = 415	
< 1.500.000 LBP	178 (42.9)
[1.500.000–3.000.000 LBP.]	119 (28.7)
[3.000.000–5.000.000 LBP.]	66 (15.9)
> 5.000.000 LBP	52 (12.5)

### Management/leadership competencies and work satisfaction

Regarding management/leadership competencies, the majority of participants **(67.5%, *n* = 80)** were aware of the detailed aspects of their work and effective at problem-solving **(65.1%, *n* = 270)**; **56.6% (*n* = 235)** immediately addressed problems when they occurred. Moreover, more than half of the participants **(54.5%, *n* = 226)** could manage people and resources. About **(60.2%, *n* = 250)** used their emotional energy to motivate others, and **(61%, *n* = 253)** enjoyed responding to people’s requests and concerns. Around **(63%, *n* = 261)** were flexible about making changes in their organizations and making strategic plans for their programs.

Regarding the satisfaction of participants with their present work, **(48.2%, *n* = 200)** considered themselves satisfied. The majority **(51.6%, *n* = 214)** were enthusiastic about their work most days, **(58%, *n* = 241)** enjoyed their work, and **(57.1%, *n* = 237)** disagreed about considering their jobs to be rather unpleasant.

The association of different baseline factors with management/leadership competencies of pharmacists was explored in the bivariate analysis. After calculating the score of management/leadership competencies (mean score = 26.8 ± 8.14), a significant association was found between management/leadership competencies and work satisfaction (*p* < 0.001), marital status (*p* = 0.002 < 0.05), and regions (*p* = 0.023 < 0.05); no significant difference was found between students and graduate pharmacists. A significant relationship was also found between work satisfaction and management/leadership competencies (*p* < 0.001), education level (*p* = 0.007 < 0.05), and monthly income (*p* = 0.033 < 0.05) (Table 3).

**Table 3:** Association of the different variables with management/leadership competencies

Variables	Management/leadership competencies, mean	*p* value	Work satisfaction	*p* value
	Mean ± SD		Mean ± SD	
*Gender, n = 415*				
Females	26.99 ± 7.99	0.474	6.98 ± 1.69	0.88
Males	26.32 ± 8.55		6.95 ± 1.76	
*Marital Status, n = 415*				
Single	27.48 ± 7.40	**0.002**	7 ± 1.73	0.493
Married-Engaged	24.53 ± 9.85		6.87 ± 1.75	
Divorced	27 ± 13.86		6 ± 2	
*Highest education level, n = 415*				
Undergraduate	27.17 ± 6.82	0.633	7.48 ± 1.59	**0.007**
Bachelor’s Degree	26.38 ± 8.85		6.96 ± 1.75	
Master’s Degree	26.12 ± 8.85		6.71 ± 1.67	
PharmD	26.45 ± 8.51		6.51 ± 1.83	
PharmD, Master’s Degree	27.94 ± 7.59		6.96 ± 1.77	
PharmD, PhD	30 ± 4.93		7.55 ± 1.59	
PhD	34 ± 0.1		6.00 ± 1.50	
*School/faculty of pharmacy, n = 415*				
Beirut Arabic University (BAU)	26.86 ± 9.69	0.485	6.88 ± 1.75	0.607
Lebanese American University (LAU)	24.82 ± 9.43		6.62 ± 1.71	
Lebanese International University (LIU)	27.35 ± 7.97		6.81 ± 1.52	
Lebanese University (LU)	26.91 ± 7.39		7.06 ± 1.75	
Saint-Joseph University of Beirut (USJ)	27.32 ± 8.72		7.02 ± 1.79	
Outside Lebanon	27.00 ± 9.29		7.08 ± 2.40	
*Region, n = 415*				
Beirut	26.65 ± 8.07	**0.023**	6.94 ± 1.90	0.821
Beqaa	23.36 ± 8.90		6.75 ± 1.17	
Mount Lebanon	27.72 ± 7.74		7.00 ± 1.71	
Nabatiyeh	29.36 ± 7.64		7.00 ± 2.10	
North	24.68 ± 9.81		7.03 ± 1.98	
South	26.54 ± 7.73		6.90 ± 1.57	
*Work sector, n = 415*				
Pharmacy	26.80 ± 7.86	0.105	7.00 ± 1.73	0.413
Pharmaceutical Company	25.00 ± 9.24		6.55 ± 1.86	
Hospital	28.70 ± 8.06		7.04 ± 1.87	
Industry	28.77 ± 7.12		7.55 ± 1.33	
Academia	26.24 ± 7.47		7.18 ± 1.42	
More than one work sector	28.42 ± 8.86		7.06 ± 1.65	
*Years of experience, n = 415*				
≤ 5	27.10 ± 7.66	0.841	6.94 ± 1.77	0.562
[6–10]	25.77 ± 9.88		7.11 ± 1.51	
[11–15]	22.59 ± 11.43		7.12 ± 1.36	
[16–20]	25.83 ± 12.38		7.67 ± 2.58	
> 20	28.11 ± 8.08		6.44 ± 1.24	
*Employment type, n = 415*				
Part-time	26.69 ± 7.49	0.793	7.10 ± 1.75	0.135
Full-time	26.90 ± 8.68		6.84 ± 1.72	
*Monthly income, n = 415*				
< 1.500.000 LBP	26.77 ± 7.49	0.089	7.15 ± 1.76	**0.033**
[1.500.000–3.000.000 LBP.]	27.27 ± 8.34		6.74 ± 1.60	
[3.000.000–5.000.000 LBP.]	25.15 ± 9.10		6.67 ± 1.76	
> 5.000.000 LBP	27.96 ± 8.47		7.21 ± 1.86	

Values marked in bold are significant *p* < 0.05

### Multivariable analysis

The linear regression taking the management/leadership competencies as the dependent variable showed a significant linear correlation between management/leadership competencies and work satisfaction, marital status, and region of living (Table 4). The correlation between management/leadership competencies and all the predictors was positive (*R* = 0.343). The R square value of 0.118 indicated that 11.8% of the variation in management/leadership competencies (dependent variable) was explained by all other factors (independent variables). In this model, work satisfaction was the variable that explained management/leadership competencies the most (Standardized coefficients Beta = 0.288). The work sector was not significantly associated with the dependent variable. With three explanatory variables in the model, the form of the regression line is: management/leadership competencies = 23.33 + (0.288 × work satisfaction) + (− 2.825 × marital status) + (− 1.873 × region).

**Table 4:** Multivariable analysis

	Unstandardized Beta	Standardized Beta	*p* value	Confidence interval	
				Lower bound	Upper bound
Model 1: Linear regression taking management/leadership competencies as the dependent variable*					
Work satisfaction	1.351	0.288	< 0.001	0.924	1.779
Marital status	− 2.825	− 0.147	0.002	− 4.575	− 1.075
Region	− 1.873	− 0.106	0.023	− 3.489	− 0.258
Model 2: Linear regression taking work satisfaction as the dependent variable**					
Management/leadership competencies	0.062	0.292	< 0.001	0.043	0.082
Education level	− 0.644	− 0.155	0.001	− 1.024	− 0.264

*Independent variables included in the model are: marital status, region, work sector, monthly income

**Independent variables included in the model are: highest educational level, employment type, monthly income, and management/leadership competencies

When considering work satisfaction as the dependent variable, the results showed that management/leadership competencies explained work satisfaction the most in this model (Standardized coefficients Beta 0.292). There was a negative association between work satisfaction and education level, B = − 0.644 (CI = − 1.024; − 0,264), where higher education levels were correlated with decreased work satisfaction. The adjusted R square value of 0.107 indicates that 10.7% of the variation in work satisfaction (dependent variable) is explained by management/leadership competencies and other independent variables. Employment type and monthly income were excluded from the model, since they were not significantly associated with work satisfaction. With two explanatory variables in the model, the form of the regression line is: work satisfaction = 6.439 + (0.062 × management/leadership competencies) + (− 0.644 × education level).

## Discussion

To our knowledge, this study is the first in Lebanon to assess the management/leadership competencies and their association with work satisfaction among a sample of Lebanese pharmacists and fifth-year pharmacy students. The sample distribution was considered of acceptable representativity, as it was overall similar to the distribution of pharmacists in Lebanon, according to the figures available from the Order of Pharmacists of Lebanon, showing a majority of women from Beirut and Mount Lebanon working in the community setting and having achieved various levels of education (a minimum of BS with or without additional degrees) (personal communication with the administration at the Order of Pharmacists of Lebanon).

Consistent with the findings of a study carried out in Dutch community pharmacies [20], work satisfaction was positively correlated with management/leadership competencies. Work satisfaction affects employees’ commitment to their jobs, which, in turn, influences their attitude to aim for higher success and development and influences their performance. Thus, if work satisfaction is supported by successful leadership, then leaders play a prominent role in promoting work satisfaction [21]. Thus, in Lebanon, pharmacists with higher leadership competencies are more satisfied with their jobs; this is also consistent with the study conducted in Texas [15]. We note that additional studies are necessary to confirm this association, due to the paucity of the literature related to it.

Regarding management/leadership competencies, our study revealed that working in a given Lebanese region affected pharmacists’ leadership/management competencies differently, which could be related to different levels of urbanism and socioeconomic status of the population, directly affecting the competencies that pharmacists need to use in their daily work [22]. Most participants reported being effective at problem-solving. While primary healthcare managers are found incompetent in problem-solving, especially in financial management [23], our findings are supported by other studies from Saudi Arabia and South Africa [24, 25]. Moreover, more than half of the participants could manage resources and people. Leaders convey high expectations to followers [26] and inspire them to become more committed and involved in realizing any vision shared in the organization. Managing human resources is the key to guaranteeing better outcomes in any organization. This result is consistent with previous findings related to management competencies among primary healthcare managers in Timor-Leste [23]. Leaders can encourage employees to succeed and do more than what is expected of them [26]. They listen, support, and attend to their needs by acting as mentors and coaches. By fostering a supportive environment for individual growth, employees become more confident and engaged at work, which ultimately improves their work satisfaction and the quality of patient care [27]. For this reason, leadership is not just for those with management positions; it should be developed in pharmacists to create successful leaders, which would advance the pharmacy practice [28, 29]. Organizations remain competitive and profitable when they have the appropriate knowledge, skills, and abilities to succeed [23].

Our findings showed that respondents’ characteristics such as work satisfaction and marital status were significantly predictive of their level of the management/leadership competencies. Being engaged or married was negatively associated with management/leadership competencies of pharmacists, likely because of the new duties added to their daily life and routine that shifted their focus from their work to other forms of complicated responsibilities acquired at home [30]. This result might also explain the fact that being single offers a broad space to enhance skills and apply the new roles of pharmacy leaders [31].

In addition, surprisingly, no significant difference in terms of leadership/management skills was found between pharmacy students and graduates, regardless of their education level. Two facts could explain this findings: either that modern pharmacy curricula are vehiculating leadership and management competencies in a way that undergraduate students are as competent as graduates with higher education or that students overestimated their competency in this domain. The first explanation could be justified by the current accreditation of all pharmacy programs in Lebanon [17], while the second can be related to a differential information bias. Further studies are necessary to further depict these findings.

Contrary to our results, there is strong evidence that a higher level of education is associated with better levels of work satisfaction as better opportunities come along [32, 33]. In our study, decreased satisfaction might be due to the lack of recognition of pharmacists’ skills and degrees in Lebanon and the fact they believe they are underpaid or even not remunerated for some services they provide [34, 35], also in line with the global study showing that satisfaction was inversely related to expectation [14]. Likewise, a previous study in Ethiopia among hospital pharmacists found that those with degree qualifications, not only diplomas, were more likely to be dissatisfied [32]. Surprisingly, a study conducted in Saudi Arabia did not show any association between education level and work satisfaction [21], where several factors were correlated with work satisfaction, including monthly income and age, but were not significant predictors of work satisfaction.

### Limitations of the study

Our study has several limitations. Its cross-sectional design does not allow for demonstrating causality. An information bias might exist, since the respondents might not have reported valid data while using an online questionnaire related to logistic difficulties. In addition, employees might have been affected by COVID-19 and the economic crisis Lebanon has been enduring lately, which might have affected the results related to work satisfaction. This situation is expected to induce a non-differential information bias, which could have driven the associations toward the null.

Although the overall sample representativeness was considered acceptable in comparison with the figures of the Order of Pharmacists of Lebanon, a selection bias might also be possible due to the same crisis context that could have affected pharmacists’ willingness to participate and the use of the snowball sampling technique among pharmacists who have internet access. Finally, although many potential confounders were taken into account, residual confounding may still exist. Future large-scale longitudinal studies are warranted to confirm our results.

Despite the limitations of the study, several recommendations can be issued based on these results: educational institutions and continuing education authorities are invited to focus on leadership and management competencies among pharmacy students and graduate pharmacists, respectively, in accordance with the core competency framework previously suggested in Lebanon [36]. At the global level, similar works can be conducted based on the frameworks recommended by FIP [37, 38]. Moreover, treating the root cause of the problem and increasing pharmacists’ satisfaction [39] would be expected to improve pharmacists’ mental health and benefit the practice and patient health [40, 41]. Although more studies are necessary to assess the factors associated with pharmacists’ satisfaction in Lebanon, these recommendations still apply at the global level.

## Conclusion

Work satisfaction and management/leadership competencies of pharmacists and pharmacy students are interrelated, although the level of satisfaction seemed lower than the declared level of competencies. These concepts are differentially affected by personal and work-related characteristics. More efforts should be exerted to improve both the satisfaction and management/leadership competencies of pharmacists in Lebanon. Further large-scale longitudinal studies are warranted to confirm our findings.

## Data Availability

The data sets used and/or analyzed during the current study are available from the corresponding author on reasonable request.
