# Correction: The association of management and leadership competencies with work satisfaction among pharmacists in Lebanon

**DOI:** 10.1186/s40545-023-00572-x

**Published:** 2023-05-12

**Authors:** Lama Zeineddine, Hala Sacre, Chadia Haddad, M. Rony Zeenny, Marwan Akel, Aline Hajj, Pascale Salameh

**Affiliations:** 1 Faculty of Pharmacy, Lebanese University, Hadat, Lebanon; 2 INSPECT-LB (Institut National de Santé Publique, d’Épidémiologie Clinique et de Toxicologie-Liban), Beirut, Lebanon; 3 School of Medicine, Lebanese American University, Beirut, Lebanon; 4 Research Department, Psychiatric Hospital of the Cross, P.O. Box 60096, Jal Eddib, Lebanon; 5 School of Health Sciences, Modern University for Business and Science, Beirut, Lebanon; 6 Department of Pharmacy, American University of Beirut Medical Center, Beirut, Lebanon; 7 School of Pharmacy, Lebanese International University, Beirut, Lebanon; 8 Laboratoire de Pharmacologie, Pharmacie Clinique et Contrôle de Qualité des Médicament (LPCQM), Faculty of Pharmacy, Saint-Joseph University of Beirut, Beirut, Lebanon; 9 Faculté de Pharmacie, Université Laval, Québec, Canada; 10 Department of Primary Care and Population Health, University of Nicosia Medical School, 2417 Nicosia, Cyprus; 11 Oncology Division, CHU de Québec Université Laval Research Center, Québec, Canada

**Correction** **:** **Journal of Pharmaceutical Policy and Practice (2023) 16:48** 10.1186/s40545-023-00554-z

Following the publication of the original article [1], the authors identified an error in the author group. The given names and family names were erroneously transposed.

The incorrect author’s names are: Zeineddine Lama, Sacre Hala, Haddad Chadia, Zeenny M. Rony, Akel Marwan, Hajj Aline and Salameh Pascale.

The correct author’s names are: Lama Zeineddine, Hala Sacre, Chadia Haddad, M. Rony Zeenny, Marwan Akel, Aline Hajj and Pascale Salameh.

The author group has been updated above and the original article [1] has been corrected.
